# Introducing Braining—physical exercise as adjunctive therapy in psychiatric care: a retrospective cohort study of a new method

**DOI:** 10.1186/s12888-023-05053-8

**Published:** 2023-08-07

**Authors:** Åsa Anger, Anna Wallerblad, Leida Kaaman, Rebecka Broman, Johan Holmberg, Tobias Lundgren, Sigrid Salomonsson, Carl Johan Sundberg, Lina Martinsson

**Affiliations:** 1 Centre for Psychiatry Research, Department of Clinical Neuroscience, Karolinska Institutet, Norra Stationsgatan 69, Plan 7, 113 64 Stockholm, Sweden; 2 Stockholm Health Care Services, Region Stockholm, Stockholm, Sweden; 3 Department of Learning, Informatics, Management and Ethics, Karolinska Institutet, Stockholm, Sweden; 4 Department of Physiology and Pharmacology, Karolinska Institutet, Stockholm, Sweden

**Keywords:** Physical activity, Exercise, Depression, Bipolar disorder, Anxiety, Psychiatry, Metabolic disease, Cardiovascular disease, Psychiatric care, Physical Activity on Prescription

## Abstract

**Background:**

Patients with severe mental disorders suffer from higher rates of poor somatic health and have shorter life expectancy than the average population. Physical activity can treat and prevent several diseases, *e.g.* cardiovascular and metabolic disorders as well as psychiatric symptoms. It is therefore of utmost importance to develop effective methods to integrate physical activity into psychiatric care. To meet this need, the physical activity intervention Braining was developed. This study aims to describe Braining, to assess the number of patients reached during the first years of pilot testing, to analyze clinical data in the group of patients participating in Braining 2017–2020 and to assess the intervention.

**Methods:**

In this descriptive retrospective study we analyzed data from all patients participating in Braining training sessions ≥ 3 times (*n* = 239), the Braining Participants. Regular patients at the clinic served as a comparison. Furthermore, medical records were studied for a smaller cohort (*n* = 51), the Braining Pilot Cohort. Data was analyzed using Chi-square and Fisher’s tests.

**Results:**

During the introduction period of Braining, 580 patients attended an information meeting about Braining, or at least one training session. 239 patients participated in ≥ 3 training sessions, considered to be participants of Braining. These Braining Participants (*n* = 239), ages 19 to 82, males 23.4%, attended between 3 and 308 training sessions (median 9). The main diagnoses were affective and anxiety disorders. Number of diagnoses ranged from 0 to 10 (median = 2). For the subsample, the Braining Pilot Cohort (*n* = 51), participants attended between 3 and 208 training sessions (median = 20). Twelve percent were working full-time, and symptom severity of depression and general anxiety was moderate. Two thirds had ≥ 3 different classes of medication. Regarding metabolic morbidity, 28% had been diagnosed with hypertension, though blood lipids, blood glucose as well as blood pressure were within the normal range. Thirty-seven percent were prescribed Physical Activity on Prescription during 2017–2020. One severe adverse event was reported.

**Conclusions:**

The Braining intervention reached all age-groups and patients with a wide and representative diagnostic panorama, suggesting that Braining could be a promising and safe method for implementing physical activity in a psychiatric patient population.

## Background

Mental illness in general is one of the leading causes of disability globally [1, 2]. Affective disorders and anxiety disorders cause major suffering and high societal costs and are common challenges in Psychiatric care [1, 2]. Individuals with depression and anxiety disorders are less physically active than average [3–5]. In depression both psychological and pharmacological treatments are recommended [6, 7]. In anxiety disorders psychological treatment is considered first-line treatment, but pharmacological treatment is also recommended [7, 8]. About 50% of those treated with cognitive behavioral therapy (CBT) for anxiety or depression recover and many others show significant clinical improvement [9]. However, access to psychological treatment is limited, mainly due to lack of resources and expertise [10–12]. Access to pharmacological treatment with antidepressant medication such as selective serotonin reuptake inhibitors (SSRIs) is sufficient in many countries; however, compliance over time is poor and these medications have well-known side effects [13]. About a third of patients do not respond to SSRIs or CBT and it usually takes weeks before response to treatment occurs [14, 15]. Therefore, in specialized psychiatric care, patients are often prescribed multiple psychopharmaceuticals [16, 17]. Of these, many have a metabolic side effect profile which contributes to impaired somatic health [18–21].

Physical activity is defined by the World Health Organization (WHO) as any bodily movement produced by skeletal muscles that requires energy expenditure is effective as prevention and treatment in many of the most prevalent somatic non-communicable diseases such as cardiovascular disease, various cancers, type 2 diabetes, osteoporosis, and obesity [19]. Physical exercise can be defined as a subset of physical activity that is planned, structured, and repetitive with the objective to improve or maintain physical fitness [22] and onwards, physical exercise will be defined by its specific attributes when described. The most physically inactive, including psychiatric patients as an important risk group, have a risk-reducing effect with only a minor increase of physical activity dosage [21]. A prospective cohort study shows that 15 min of daily moderate intensive aerobic physical activity could extend life expectancy by three years [23]. Additionally, physical activity has very few side effects.

The WHO’s general recommendation for physical activity for adults are also applicable for patients with depression [19]. Additionally, for mild to moderate depression there is growing evidence for effects of physical activity [24]. Studies show a comparable reduction in depressive symptoms to treatment with antidepressants or psychotherapy [25–31]. Physical exercise is not only effective as treatment of mild to moderate depression. In moderate to severe depression there are clinical as well as molecular studies, indicating that physical exercise as add- on to pharmacological treatment could have a positive synergistic effect [26, 32]. However, there is still a lack of studies regarding physical exercise and physical activity of clinically well characterized psychiatric patients compared to the load of research done on CBT and SSRIs for instance. Furthermore, the risk of developing or relapsing into depression, as well as developing anxiety symptoms or disorders is reduced by regular physical activity as well as reduced time spent sedentary [31, 33–35].

Despite the growing evidence of the effects of physical activity on psychiatric symptoms, it remains to define what the specific mode, intensity and duration should be for optimal effect [36]. Some studies have shown no differences in effect on depression between aerobic and strength training or between exercise conducted at different intensities [25, 37]. Other studies have shown greater effects of aerobic training performed at moderate to high intensity level compared to low intensity activities [27, 31, 38].

Physical activity has shown an anxiety reducing effect both acutely and long-term [39, 40]. Though the research available is limited, physical activity is recommended as an add-on treatment [41]. In several studies [41–43], occasional exercise sessions of vigorous intensity could reduce the risk of panic attacks in patients with panic disorder. In accordance with those findings, patients with generalized anxiety disorder experienced an improvement in anxiety symptoms and feelings of energy directly after vigorous intensity exercise [18, 41, 44]. Regular physical activity can reduce symptoms in people with anxiety symptoms or anxiety disorders [18, 24, 45]. Physical activity has anxiety-reducing effects and better long-term effects than placebo, but significantly less effects than CBT or pharmacological treatment [45, 46]. Regarding bipolar disorder, there are few RCTs investigating physical exercise as part of lifestyle interventions. However, the main outcome in these studies have been metabolic changes, rather than mental health improvement [47–49].

Despite the clear positive effects of physical activity on symptoms and somatic side-effects in psychiatric patients, it is not yet implemented in the normal treatment range in psychiatric care [4, 25, 50, 51]. Physical Activity on Prescription has been scientifically studied [52] and is considered implemented as a method for promoting physical activity for patients in Sweden. This has led to an ongoing project cofounded by the European Union aiming to spread Physical Activity on Prescription in several countries in Europe [53]. A recent report from the National Board of Health and Welfare notes 5–10 Physical Activity Prescription/1000 patients visits per year in primary care in Sweden [54], but there is a lack of studies on Physical Activity on Prescription in psychiatric care as well as for patients in primary care with psychiatric disorders.

Due to the clear health benefits of being physically active, there is a need to develop a structured physical activity method that can be implemented and integrated into everyday psychiatric care. Therefore, in 2017, we developed Braining, a structured clinical intervention to support patients to initiate and execute moderate to vigorous physical activity, at a large psychiatric clinic in Region Stockholm. This study aims to retrospectively describe the method, the participating patients, and their participation in training sessions during the first four years.

## Methods

### Aims

This study aims to describe the method Braining, to assess the number of patients reached by the intervention during the first years of implementation, to analyze clinical data in the group of patients participating in Braining during the initial years 2017–2020 regarding medical and demographic variables, and to assess the intervention in terms of participation in training sessions, as well as any adverse events.

### Participants and setting

Patients at two outpatient units (the Affective Outpatient Unit, and the Affective, Anxiety, and Trauma Outpatient Unit, n≈2 100 patients) at a large psychiatric clinic (n≈11 000 patients) in Region Stockholm, Psychiatry Southwest were the main target group for Braining during the study period 2017–2020. The patients at these units were predominantly patients with bipolar disorders, depression, anxiety disorders and post-traumatic stress disorder (PTSD). To a minor extent, patients from other units with depression, anxiety, sleep disturbance or stress as sub-symptoms of other psychiatric disorders, were also allowed to participate. Patients were mainly participating when in outpatient care but could also participate while subjected to inpatient care.

During 2017–2020, 580 patients participated in Braining on any occasion of which 566 were available to be contacted and were included in the study. Of these, the 239 patients that participated in three or more training sessions were invited to participate in long-term follow-up including a medical record review. Onward in this text, this group of 239 patients are called the Braining Participants. 51 patients that met the inclusion criteria and not the exclusion criteria agreed to participate, namely the Braining Pilot Cohort.

**Fig. 1:**
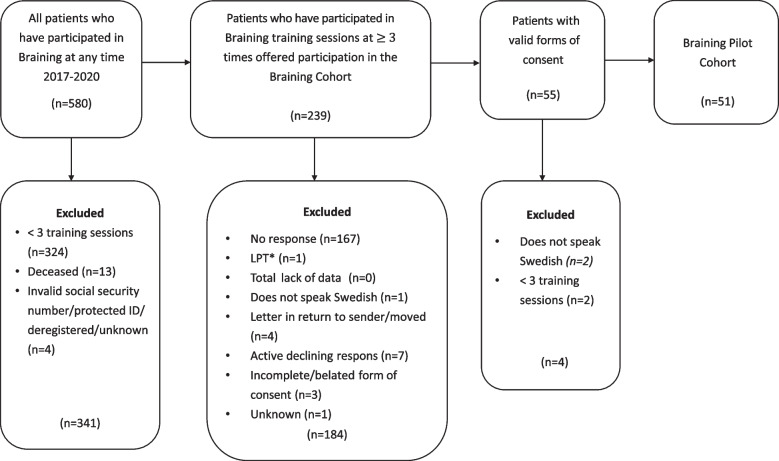
Flow chart. Overview of participation process, detailing number of included and excluded patients. *LPT: care in accordance with the Compulsory Psychiatric Care Act (Lagen om psykiatrisk tvångsvård, LPT)

Inclusion criteria: all participants with three or more training sessions in total. Exclusion criteria: participants deceased at time for inclusion (*n* = 13), lacking baseline data (*n* = 0), difficulty speaking or understanding the Swedish language (*n* = 3), cared for in accordance with the Compulsory Mental Care Act (Lagen om psykiatrisk tvångsvård) (*n* = 1) at the time of inclusion. Difficulty speaking or understanding the Swedish language was defined as needing translator services to communicate with staff. Also excluded were participants having an invalid social security number, protected identity, invitation letter in return to sender/moved, incomplete or belated form of consent or unknown (*n* = 8) (Fig. 1).

### Design and procedure

The study is a retrospective, descriptive cohort study. Data were retrieved from available medical records, as well as from the central register at the Health Care Services Stockholm County (Stockholms Läns Sjukvårdsområde). Patients who fulfilled the inclusion criteria (*n* = 239) were sent written information about the study along with a written consent form. Those who did not respond in writing, were reminded by follow-up letters at up to two times. They had the opportunity to ask questions via a telephone contact number. Out of these, 51 patients did not meet the exclusion criteria, agreed, and were included in the cohort planned for long-term follow-up and medical record review (Fig. 1).

For all participants in Braining, data was collected on an individual level from the central register at the Health Care Services Stockholm County (Stockholms Läns Sjukvårdsområde). In attempt to visualize representativity, the patient group enrolled at the two outpatient units (the Affective Outpatient Unit, and the Affective, Anxiety, and Trauma Outpatient Unit) was used as reference group and data was only available on a group level.

The same information was obtained, on the same central register level for the Braining Pilot Cohort (*n* = 51) with written consent to medical record review and long-term follow-up.

### The Braining method

Since 2017, patients at Psychiatry Southwest in Region Stockholm, have been offered Braining, in order to initiate, support and execute physical exercise regularly. This novel method per se has not yet been validated, though it aims to follow the recommendations for physical activity from the WHO [19] when it comes to intensity, duration and recommended frequency. Braining is a structured clinical intervention, with core components of repeated 30–45 min moderate to vigorous intensity group physical exercise sessions. The Braining physical exercise sessions consist of a 5 to 7 min long warm up followed by 20 to 30 min of interval based aerobic movements e.g., jogging or jumping jacks as well as body weight strength exercises e.g., squats and pushups. The sessions end with a short cool down with mobility or balance focus. The sessions are supervised by two trained members of the psychiatric staff, one *instructor* and one *host*. The *instructor* is teaching the session and thereby showing correct technique and intensity. The *host* supports the participants and gives them individual adjustments during the class. To ensure quality and consistency, the *instructor* and *host* use the Braining Box, a physical and digital tool with photos, written instructions and filmed exercises as well as complete classes, developed by physiotherapists, psychiatrists and employees at the clinic. The material in the Braining Box is publicly available [55]. The Braining instructors use the Borgs scale of rated perception of exertion [56] to mediate eligible intensity level between 11 and 17. Whenever participants appear to exceed 17 or perform under the limit of 11, the Braining leader will encourage them to adapt. Since this was a clinical project, the intensity was not controlled for with any other tools at this stage. Physical exercise is accompanied by continuous short follow-ups and support to promote compliance, all led by educated and licensed psychiatric staff. Before and after the intervention period, measurements and evaluations are performed. Physical exercise is added to treatment as usual, included in the patient care plan, and covered by the regular healthcare fee. Each physical exercise session is preceded by a short individual visit with staff for a brief assessment of mental and physical status and for motivational support.

The goal for participation when introducing Braining is three physical exercise sessions/week during a three-month period, but patients participate voluntarily. Each participation in the structured program begins and ends with a motivational and educational visit, provided in a group seminar and/or an individual visit. In conjunction with this, participants are also offered a mental and physical examination, assessment scales on symptoms and quality of life, and the ability to submit blood samples. The main target group during the study period was patients with predominantly affective disorders or anxiety disorders in outpatient care, but patients with other diagnoses or inpatient care were also allowed to participate.

### Variables

The burden of disease and level of function was measured regarding psychiatric diagnoses, medical use, psychiatric care visits, and occupational status. For all patients, namely the reference group (*n* = 2 144), the Braining Participants (*n* = 239), and the Braining Pilot Cohort (*n* = 51), data was collected regarding age, gender, and psychiatric diagnoses. For the reference group, data was only available on a group level.

### Braining participants

The patients participating in three or more training sessions (*n* = 239) in 2017–2020 are described in terms of age, gender, ICD-10 (International Classification of Diseases) psychiatric diagnoses, number of training sessions, and number of contacts with psychiatric health care in outpatient units, emergency units and inpatient care. Data on training sessions was accessible through the Swedish Classification System of Care measures (Klassifikation av vårdåtgärder), where every group training session was coded with a specific Braining group session code (QV011). This data could not be related to individuals and was obtained for the different subgroups separately, to enable statistical analyses.

Information about the patient group enrolled in total (*n* = 2 144) at the two outpatient units, used as reference group, were available on a general level, which was also used for comparison with the group that were reached by the structured intervention Braining.

### Braining pilot cohort

For the Braining Pilot Cohort (*n* = 51) more detailed data was available and extracted from medical records, in addition to description of age, gender, number of training sessions, and number of contacts with psychiatric health care in outpatient units, emergency units and inpatient care, as for the Braining Participants.

Additional data included psychiatric and somatic diagnoses, level of function in terms of degree of sick leave/occupational status, symptom level based on self-assessment scales for symptoms of depression in terms of PHQ-9 [57] and anxiety in terms of GAD-7 [58], self-assessed health-related quality of life in terms of EQ-5D-5L/EQ-5D-3L [59], clinician assessed severity of psychopathology in terms of CGI-S [60, 61] as well as psychopharmaceutical treatment classified by ATC (Anatomical Therapeutic Chemical Classification System) code, blood pressure, molecular parameters (blood lipids, blood glucose).

PHQ-9 (9-item Patient Health Questionnaire-9) is a questionnaire designed to screen for depression in medical settings. Its sensitivity to detect major depressive disorder has been thoroughly tested and has yielded good results. The total score ranges from 0 to 27 and the standard cut-off score to detect possible major depression is 10 or above. 10–14 constitutes a diagnostic grey area and 15–27 indicates the existence of major depressive disorder [57].

GAD-7 (7-item Generalized Anxiety Disorder Questionnaire) is one of the most frequently used measures for anxiety, because of its validity and diagnostic reliability. It has been deemed sensitive to detect changes in anxiety severity over the course of treatment, with a minimal clinically important difference equal to 4. The seven items of the questionnaire describe the seven core symptoms for generalized anxiety disorder and asks how often participants have experienced these symptoms within the last two weeks. Each item is scored as 0–3 with 0 translating to not at all and 3 to almost every day. The total score ranges from 0 to 21, with 5 constituting the threshold value for mild anxiety, 10 for moderate and 15 for severe anxiety [58].

CGI (Clinical Global Impression Scale) [62] is a widely used tool in psychiatric clinical practice designed to rate the patient’s severity of symptoms (CGI-S), improvement (CGI-I), and the effectiveness of a specific treatment (CGI-E) [60]. The present study used the CGI-S, severity of symptoms measure. Several studies evaluating the reliability and validity of CGI have been published, also with adjustments to bipolar disorders [63] but results are mixed, and no established psychometric properties exists. Promising studies has shown sensitivity to change, significant correlations to other clinical routine outcome measures, and satisfactory interrater reliability [63, 64].

EQ-5D-5L is a well-known, reliable and valid instrument measure of an individual’s health-related quality of life. It has also been deemed relatively responsive to changes in health status. The questionnaire consists of five dimensions; mobility, self-care, usual activities, pain/discomfort and anxiety/depression. Each dimension has five response categories ranging from no problems to severe/extreme problems, which creates a total of 3125 unique health states. This generates an index value of -0,285 to 1,00, where 1,00 constitutes perfect health, 0 is a state equal to death and negative values constitute a state worse than death. The measure is administered jointly with a visual analogue scale (VAS), where participants rate their current health state on a scale of 1 to 100. EQ-5D-3L is the preceding version of the instrument, used clinically during the first part of the present study period, thus exchanged clinically along the way when the instrument was updated [59].

### Time measurement points

**Fig. 2:**
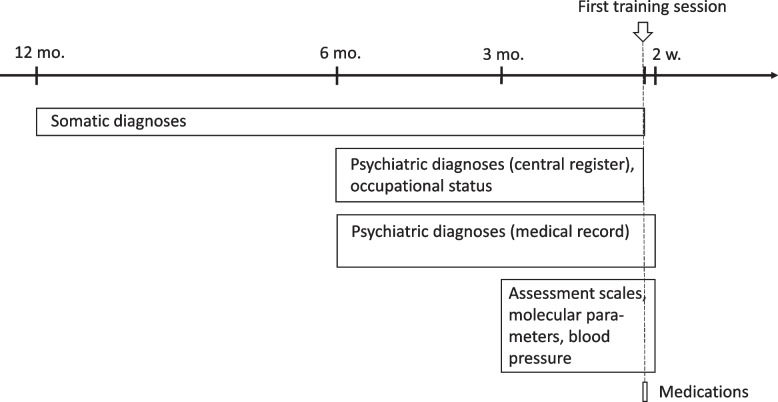
Time measurement points

The time measurement points used were defined in relation to the first occasion the patient took part in a training session within the Braining intervention. Due to the naturalistic setting, and the retrospective design, minor adjustments were made in order not to lose data that for example were dated a week outside the originally planned measurement point. Therefore, an additional 2 weeks were added to the time frames for diagnoses, molecular parameters, blood pressure and assessment scales (Fig. 2).

### Method assessment

The participation in Braining in terms of number of training sessions is described for both groups, as well as any adverse events.

### Statistical analysis

#### Descriptive statistics

All variables were summarized and tabulated for the three separate groups Reference group, Braining Participants, and Braining Pilot Cohort, respectively. Categorical variables were described with frequencies (n) and percentages (%). Continuous variables were described with medians or means, and standard deviations.

The background variables describing the Reference group included: age, gender and psychiatric diagnoses. For the group Braining Participants, additional variables were available: number of contacts with psychiatric health care in outpatient units, emergency units and inpatient care, and number of training sessions. Finally, for the Braining Pilot Cohort, further additional variables were available: both psychiatric and somatic diagnoses, level of function in terms of degree of sick leave/occupational status, symptom level based on self-assessment scales for symptoms of depression and anxiety, self-assessed health-related quality of life, clinician assessed severity of psychopathology as well as psychopharmaceutical treatment classified by ATC (Anatomical Therapeutic Chemical Classification System) code, blood pressure and molecular parameters.

Description of the different groups were presented with tabulation of diagnostic spectra. In addition, number of diagnoses and number of medications were presented graphically with bar plots.

The feasibility of the Braining intervention was evaluated with description of the different groups and observation of the actual participation of patients in training sessions. Participation was partly visualized in a bar plot for Braining Participants to show frequency distribution, see Fig. 1. Additionally, number of training sessions were tabulated for Braining Participants and Braining Pilot Cohort.

#### Comparison between groups

The variables of diagnostic spectra, number of training sessions, outpatient visits, emergency visits and inpatient care were summarized and tabulated for Braining Participants and Braining Pilot Cohort to enable comparison.

Specific data on similarities and differences between groups could inform on the ability of the Braining intervention to reach out to different patient groups, and also inform on research questions for further studies. Notably, the clinical nature of study design does limit the conclusion possible to make from observed differences and similarities between groups.

#### Significant statistical differences between groups

Significant testing of group differences between Braining Participants and Braining Pilot Cohort was performed for the variables of gender and age. Differences between groups on variable of gender was evaluated with Chi-squared tests. Single cells in the categorical variable of age groups included less than five subjects so data did not meet the assumptions of Chi-squared test. Instead, Fisher’s exact test was chosen to evaluate differences in age distribution.

All data analysis was conducted using R [65], the tidyverse package [66] version 1.2.0 and the tableone package [67] version 0.13.2.

## Results

In a naturalistic, clinical setting at two outpatient units during the first phase of implementation, 580 patients attended an information meeting about Braining, or at least one training session, during the first four years. 239 patients participated in ≥ 3 training sessions, here called the Braining Participants. In 2021, these were invited in writing to participate in the Braining retrospective study, 55 patients accepted and gave informed consent. Out of these 51 did not meet the exclusion criteria and could be included in the planned cohort, here called the Braining Pilot Cohort (Fig. 1).

### Braining participants

**Fig. 3:**
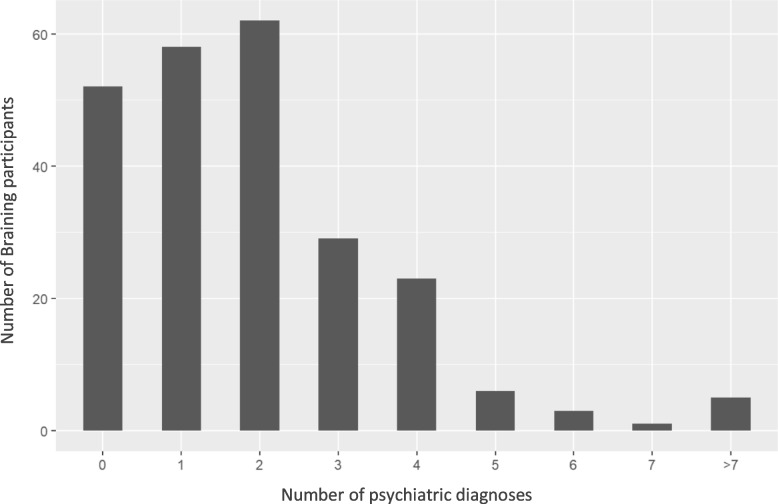
Number of psychiatric diagnoses among Braining Participants. Distribution of number of diagnoses per participant. We noted that 30 participants (12.6%) did not have a diagnose in the data material. An additional 22 participants (9.2%) only had a diagnose for examination or observation (ICD-10 codes Z00.4 and Z03.2). Note that listed diagnoses above show the total number of diagnoses and does not represent individual patients

Among the Braining Participants (*n* = 239), ages ranged from 19 to 82. There was a significant difference in age composition; 8.4% were in the age group 20–30 compared to 18.3% in the reference group. There was also a significant difference regarding gender, where 23.4% were male compared to 32.7% in the reference group. The main diagnoses in the reference group were represented, namely depression, bipolar disorders, anxiety disorders, PTSD, attention deficit hyperkinetic disorder (ADHD) and autism. Number of diagnoses ranged from 0 to 10 (median = 2) (Tables 1 and 2 and Fig. 3).

**Table 1:** Background characteristics, gender, age, diagnostic groups. Braining Participants compared to the reference group

	**Braining Participants, n (%)**		**Reference group, n (%)**	
N	239		2144	
Male	56 (23.4)		701 (32.7)	
Age				
18–20 years	2 (0.8)		38 (1.8)	
21–30 years	20 (8.4)		392 (18.3)	
31–40 years	56 (23.4)		481 (22.4)	
41–50 years	53 (22.2)		456 (21.3)	
51–60 years	61 (25.5)		445 (20.8)	
61–70 years	29 (12.1)		246 (11.5)	
71–80 years	17 (7.1)		157 (7.3)	
81–90 years	1 (0.4)		38 (1.8)	
> 91 years	0 (0.0)		4 (0.2)	
Missing	0 (0.0)		5 (0.2)	
**Diagnoses**	**n**	**Ratio diagnose/n**	**n**	**Ratio diagnose/n**
Bipolar disorder (F31)	63	0.26	962	0.45
Depressive disorders (F32 + F33)	85	0.36	657	0.31
Anxiety disorders (F40 + F41 + F42)	65	0.27	602	0.28
Stress reactions and PTSD (F43)	42	0.18	460	0.21
Autism (F84)	8	0.03	121	0.06
ADHD (F90)	22	0.09	331	0.15

The reference group consists of the patient group at the Affective Outpatient Unit, and the Affective, Anxiety, and Trauma Outpatient Unit. For all groups, age is attained age 2019. There was a smaller proportion of men among the participants compared to the reference patients (χ2(1, *N* = 2144) = 8.09, *p* = .004). We also found a different age distribution among the participants compared to the reference patients (Fisher’s exact test, two-tailed, *p* = .009). Diagnostic groups classified according to ICD-10, version 2019. Note that listed diagnoses above show the total number of diagnoses, i.e. one individual can contribute with more than one diagnosis. Comparison between groups were therefore not possible. A relative frequency of the number of diagnoses in respective group could still give relevant information to compare groups, therefore reported above

**Table 2:** Descriptives of the Braining Participants compared to the Braining Pilot Cohort, diagnostic spectra

Diagnoses	Braining Participants, n (%)	Braining Pilot Cohort, n (%)
N	239	51
Male	56 (23.4)	13 (25.5)
Bipolar affective disorder	63 (26.4)	19 (37.3)
Depressive disorder	76 (31.8)	21 (41.2)
Social phobia	8 (3.3)	3 (5.9)
Panic disorder	12 (5.0)	5 (9.8)
Generalized anxiety disorder	19 (7.9)	5 (9.8)
Anxiety disorder, unspecified	17 (7.1)	4 (7.8)
Obsessive–compulsive disorder	5 (2.1)	3 (5.9)
Posttraumatic stress disorder	32 (13.4)	5 (9.8)
Reactions to stress and adjustment disorder	10 (4.2)	10 (19.6)
Substance use disorder	8 (3.3)	3 (5.9)
Personality disorder	7 (2.9)	1 (2.0)
Autism	8 (3.3)	5 (9.8)
Attention deficit hyperkinetic disorder (ADHD)	22 (9.2)	6 (11.8)
Number of psychiatric diagnoses median [min, max]	2.00 [0.00, 10.00]	

Diagnoses according to ICD-10, version 2019. Note that listed diagnoses above show the total number of diagnoses, i.e. one individual can contribute with more than one diagnosis. For the Braining Participants the diagnoses are derived from central registers, for the Braining Pilot Cohort from medical chart review

**Fig. 4:**
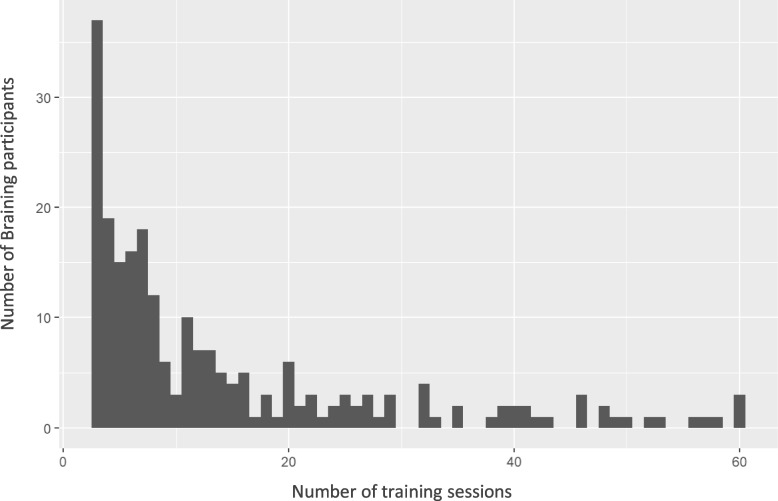
Braining Participants, number of training sessions. Of the participants (*n* = 239), 13 exercised more than 60 times, 5 exercised more than 150 times. These frequent participants participated 85, 90, 91, 98, 103, 124, 131, 146, 158, 207, 208, 232, 308 times, respectively

The amount of individual training sessions varied from 3 to 308 (median = 9) for the most active participant. 13 participants exercised more than 60 times, 5 exercised more than 150 times (median = 9). In total, this rendered 5235 training session visits (Table 3 and Fig. 4).

**Table 3:** Descriptives of the Braining Participants and Braining Pilot Cohort extended^a^, outpatient visits, emergency visits, inpatient care, training sessions

	Braining Participants, median [min, max]	Braining Pilot Cohort extended^a^, median [min, max]
N	239	55
Training sessions	9 [3,308]	20 [3,208]
Outpatient visits (excl. training sessions)	53 [0,313]	45 [0, 313]
Emergency visits	2 [0, 66]	1 [0, 41]
Hospitalizations	1 [0, 27]	0 [0, 17]

^a^Braining Pilot Cohort (*n* = 51) plus 4 patients (*n* = 55) that were later excluded during medical record review, included here due to lack of individual data for these variables

The number of outpatient visits (physical, video and telephone), training sessions excluded, varied from 0 to 313 (median = 53). In total this rendered 14,960 outpatient visits. The number of emergency visits varied from 0 up to 66 (median = 2). The number of hospitalizations ranged from 0 up to 27 (median = 1) (Table 3).

### Braining pilot cohort

Data from medical records were available for the Braining Pilot Cohort (*n* = 51). Ages ranged from 21 to 75. The main diagnoses in the reference group were represented in this group as well (depression, bipolar disorder, autism, ADHD, PTSD, general anxiety disorder, panic syndrome, social phobia, and obsessive–compulsive disorder) (Table 2).

The amount of training sessions varied from 3 up to 208 for the most active participant (median = 20). In total, this rendered 1 766 individual training session visits (Table 3 and Fig. 4). The number of outpatient visits (physical, video and telephone), training sessions excluded, varied from 0 to 313 (median = 45). The number of emergency visits varied from 0 up to 66 (median = 2). In total this rendered 2 913 outpatient visits. The number of hospitalizations ranged from 0 up to 27 (median = 1) (Table 3). Data concerning training sessions and health care contacts includes 4 additional participants that were later excluded during medical record review (Fig. 1). Out of the group, 37.3% were prescribed Physical activity on Prescription (Table 4).

**Table 4:** Descriptives of the Braining Pilot Cohort; metabolic parameters, psychiatric assessment scales, somatic diagnoses, occupational status, neuropharmaceuticals

	Braining Pilot Cohort
N	51
Metabolic parameters, mean (SD)	
fP-HDL-cholesterol	1.40 (0.42)
fP-LDL-cholesterol	3.37 (0.94)
fP-cholesterol	5.34 (1.01)
fP-triglycerides	1.54 (0.77)
P-glucose	5.80 (0.98)
Systolic blood pressure	128.07 (17.09)
Diastolic blood pressure	81.37 (11.31)
Psychiatric assessment scales, mean (SD)	
PHQ-9	12.55 (8.16)
GAD-7	10.45 (5.80)
CGI-S	3.42 (1.44)
EQ-5D-5L	0.64 (0.21)
EQ-5D-3L	0.52 (0.27)
EQ 5D general health	47.98 (26.35)
Prevalent somatic diagnoses, n (%)	
Diabetes	3 (5.9)
Hyperlipidemia	3 (5.9)
Hypertension	14 (27.5)
Asthma	3 (5.9)
Pain conditions	18 (35.3)
Cancer	7 (13.7)
Occupational status, n (%)	
Full-time work	6 (11.8)
Sick-leave, full- or part-time	32 (62.7)
Retirement	10 (19.6)
Missing	3 (5.9)
Neuropharmaceuticals (by ATC code), n (%)	
Antipsychotics (exkl lithium)	25 (50.0)
Lithium	14 (27.5)
Antidepressants	33 (64.7)
Anxiolytics (excl. bensodiazepines)	2 (3.9)
Bensodiazepines	11 (21.6)
Hypnotics and sedatives (excl. bensodiazepine derivates and benzodiazepine related drugs)	15 (29.4)
Bensodiazepine derivates and benzodiazepine related drugs	22 (43.1)
Psychostimulants	5 (9.8)
Antiepileptics	13 (25.5)
Antihistamines	17 (33.3)
Opioids	4 (7.8)
Other	
Physical Activity on Prescription (%)	19 (37.3)

On a group level, depression, and general anxiety symptom severity (PHQ-9, GAD-7) were moderate. Clinician assessed evaluation measure of severity of psychopathology (CGI-S) was mild to moderate. Data on occupational status showed that 11.8% were working full-time, 62.7% were on full- or part-time sick-leave, and 19.6% were retired. Regarding somatic morbidity, 27.5% had been diagnosed with hypertension, and 35.3% with a pain condition. Blood lipids, blood glucose as well as blood pressure were within the normal range (Table 4).

**Fig. 5:**
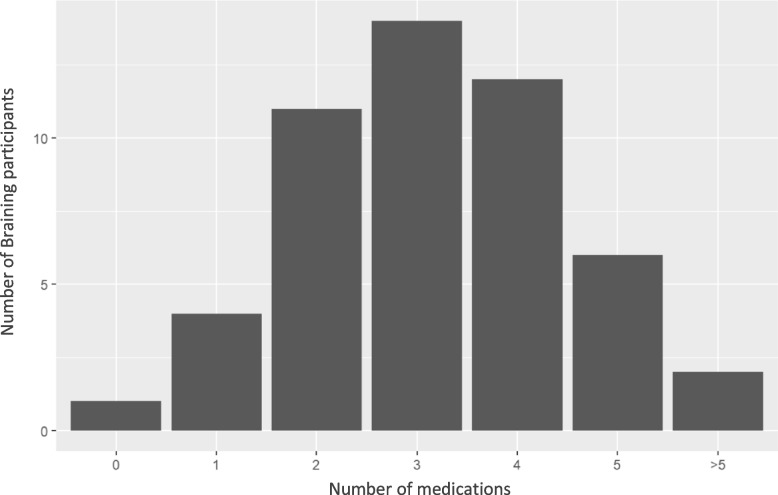
Braining Pilot Cohort, number of medications. Braining Pilot Cohort (*n* = 51). Medications classified according to ATC code

Two thirds had ≥ 3 different classes of medication (Fig. 5). 64.7% of the patients had treatment with antidepressants, 50% with antipsychotics, 27.5% with lithium, 25.5% with antiepileptics, 33.3% with antihistamines. 21.6% were treated with benzodiazepines, 43.1% with benzodiazepine-related sleep medications and 8% were treated with opioids (Table 4).

Between 2017 and 2020, two adverse events were reported. First, one patient with a previously known somatic condition had a serious event during a training session. Second, an administrative difficulty to register an inpatient for a training session, resulted in the patient leaving the premises without permission, and later returning safely to the ward. The former was classified as a serious adverse event.

## Discussion

This is the first study to describe the method Braining, a structured clinical intervention led by trained psychiatric staff, with core components of 30–45 min of moderate to vigorous intensity group physical exercise sessions accompanied by continuous short follow-ups and support to promote compliance.

The main finding in the present study was that the intervention Braining, was able to introduce physical exercise to a large group of psychiatric patients in a psychiatric outpatient setting. The population was patients in need of psychiatric outpatient care, with a variety of symptom severity and a few severely ill inpatients. The core components of the method offered 5235 training session visits as add on to their on-going treatment as usual, suggesting that patients were offered closer contact to the clinic during this period of their care compared to before Braining was introduced. Another important finding was that Braining could be implemented in ordinary psychiatric care without adding external resources. Moreover, during the observed time frame, very few adverse events were reported, suggesting an adequate level of safety.

Additionally, Braining is a new and integrated clinical method that might be more accessible to clinics with limited resources. The Braining intervention, executed on the premises of the clinic and with psychiatric staff as participating supervisors, has similarities as well as unique features compared to other physical exercise interventions in previously published studies. The main focus of aerobic physical exercise, aiming to reach the WHO guidelines [19] is in line with a majority of studies [68, 69]. Previous studies of physical exercise interventions have had both supervised and unsupervised physical exercise sessions. However, in those studies, mainly external physical exercise specialists such as physiotherapists and trainers [68] supervised the sessions. Therefore, this type of method might serve in situations with limited resources. Furthermore, since psychiatric staff take an active part in the treatment, there are possible occupational health benefits, which would be in line with previous studies on physical activity among health care staff [70], and could possibly improve caregiver and patient relationship.

Moreover, an important finding was the burden of disease and ongoing advanced treatment in the patients participating in Braining during the study period, indicating that the method reached a group who benefits the most from physical exercise. Almost one third of the participants had a hypertension diagnosis, but on a group level, blood pressure was registered during the intervention period within the normal range. During medical record review, we noticed that 6 out of 51 individuals had an elevated blood pressure, defined as a single measured value of ≥ 140/90 mmHg. This could be comprehended as well-treated blood pressure in this population.

Only 10% of the group were working full-time, and the amount of regular outpatient visits, emergency visits and in-patient care during the studied period was considerable. Two thirds of the group had ≥ 3 different classes of psychopharmacological medications. Altogether, this could indicate that the burden of disease in the group was substantial. However, the symptom level based on self-assessment scales on a group level was moderate. A possible explanation could be that Braining probably was introduced more often when patients were past the most acute phase symptom wise. The fact that training sessions were scheduled during daytime, could also have favored patients without full-time occupation.

As expected in this clinical population, participation frequency varied greatly. Some patients participated actively over several years, accumulating hundreds of physical exercise sessions, while others participated in a limited number of sessions. For this reason, with the present study design it is impossible to draw conclusions concerning the amount of Braining physical exercise sessions that the fully implemented Braining method might lead to. In a large-scale Swedish RCT comparing physical exercise with treatment as usual for depression, 31.7% attended no exercise sessions at all and 39.6% attended 12 or more sessions during the 12-week intervention period [37]. This indicates a large variation in participation/execution in this type of physical exercise intervention. Another study using running as add-on treatment for patients with severe depression in psychiatry reported a decline rate of 40% among eligible patients due to lack of interest or time and a drop-out of 55% at 6 months in included participants [71], altogether indicating that studies in this population are challenging to conduct.

From March and onwards during 2020, the Covid-19 pandemic greatly affected the healthcare system. Psychiatry Southwest continued to offer Braining as outdoor group training sessions, while most group therapy sessions and many physical visits were cancelled in psychiatric care in Sweden and even more so in other countries. This was not at focus in the present study, but as expected, the clinical impression was that the participating rate was lower during 2020 than in 2017–2019, mainly due to pandemic restrictions.

Another important finding of this study was the low number of adverse events, with only two such events reported during the first four years. A review and meta-analysis of exercise intervention studies concluded that the risk of serious adverse events was not elevated, but that the relative risk of minor adverse events was increased [72]. As in this retrospective study, adverse events were often not defined prior to the study and the ways of reporting minor adverse event were not clearly defined. However, in the long run, the positive health aspects from physical activity outnumber the eventual minor health risks [18, 19].

Another interesting finding was that that 37,3% of the Braining Pilot Cohort were prescribed Physical Activity on Prescription. Compared to the rest of the clinic Psychiatry Southwest, and even more so compared to primary care patients in Sweden [54], this is a substantial proportion. The result indicates that the implementation of Braining might lead to raised awareness regarding the importance of physical activity in the clinic. Studies investigating the implementation of Physical Activity on Prescription in psychiatry are scarce. A survey among forensic psychiatry staff in Sweden indicates that 50% of staff use Physical Activity on Prescription at least occasionally [73].

### Strengths and limitations

The prerequisites for this observational study led to several limitations affecting the outcome of data analyses. At the outset, no plans were made in preparation for a research project. However, since the participation rate during the first years was unexpectedly high, in addition to clinical observations regarding participant morbidity, a decision was made to conduct a retrospective study. As a result of this naturalistic setting, clinical data collection was incomplete. A participant could, for example, have participated in sessions prior to submitting start-up evaluations and measurements, which the set time variable did not cover. Furthermore, training sessions, submission of blood samples and other measurements were not mandatory. Regarding results of diagnoses in the reference group, there was a certain overlap in data, meaning that one patient could contribute with several data points in the same diagnosis group. For this reason, data in Table 1 is presented with a larger margin of error. Yet another limitation that affected evaluation of the implementation was our definition of patient participation as number of registered sessions in patient medical records. In other words, there were no objective measurements nor self-report inventories to ensure that participants attained the intended level of exertion during the sessions. At the same time, the naturalistic setting offers a well-needed perspective on the challenges of intervention implementation in a clinical setting with a substantial rate of high morbidity. This study could therefore provide a foundation for study design and hypothesis generating of upcoming research projects.

### Future studies

In order to evaluate the effects on patients´ mental and physical health as well as the method´s feasibility in a clinical setting, additional studies are needed. Some are already planned, such as long-term follow-up interviews with the clinical Braining Pilot Cohort (*n* = 51). The main focus will be patients´ experience of Braining, as well as long-term effects of participation. Additionally, we will enquire about minor adverse events that were not previously reported. Controlled feasibility studies that measure change in physical exercise (Braining sessions as well as other forms of physical exercise) are necessary, preferably using both objective measures such as accelerometer and validated questionnaires. A pilot study followed by a multicenter RCT studying the effects on patients, the implementation process and the experience end possible health effects on the participating staff are planned in this manner. In order to explore the compliance, effect, implementation, and patient experience of Physical Activity on Prescription in psychiatry, further studies on Physical Activity on Prescription in psychiatric care are needed.

## Conclusions

This retrospective study shows that a new structured physical exercise intervention called Braining has reached patients in all age-groups and with a wide and representative diagnostic panorama, indicating that Braining might be a promising and safe method to implement physical activity in a psychiatric setting. We suggest that the method is evaluated in future clinical trials, in regards of feasibility, effects, cost effectiveness and experiences from patients and personnel.

## Data Availability

The datasets generated and analyzed during the current study are not publicly available due to individual privacy even though pseudonymized but are available from the corresponding author on reasonable request.

## Abbreviations

ATC: Anatomical Therapeutic Chemical Classification System
ADHD: Attention Deficit Hyperactivity Disorder
CBT: Cognitive behavioral therapy
EQ5D-3L, EQ5D-5L: EQ5D™ is a trademark of the EuroQol Group, a self-assessment instrument for describing and valuing health status
GAD-7: Generalized Anxiety Disorder 7 Items
ICD-10: International Classification of Diseases and Related Health Problems, 10th Revision
PHQ-9: Patient Health Questionnaire9 Items
PTSD: Post-traumatic stress disorder
RCT: Randomized Controlled Trial
SSRI: Selective serotonin reuptake inhibitors
WHO: World Health Organization
