# Correction: Introducing Braining—physical exercise as adjunctive therapy in psychiatric care: a retrospective cohort study of a new method

**DOI:** 10.1186/s12888-024-05775-3

**Published:** 2024-05-03

**Authors:** Åsa Anger, Anna Wallerblad, Leida Kaaman, Rebecka Broman, Johan Holmberg, Tobias Lundgren, Sigrid Salomonsson, Carl Johan Sundberg, Lina Martinsson

**Affiliations:** 1 Centre for Psychiatry Research, Department of Clinical Neuroscience, Karolinska Institutet, Norra Stationsgatan 69, Plan 7, Stockholm, 113 64 Sweden; 2 Stockholm Health Care Services, Region Stockholm, Stockholm, Sweden; 3 Department of Learning, Informatics, Management and Ethics, Karolinska Institutet, Stockholm, Sweden; 4 Department of Physiology and Pharmacology, Karolinska Institutet, Stockholm, Sweden

**BMC Psychiatry (2023) 23:566**

10.1186/s12888-023-05053-8

Following publication of the article, an error in the Abstract came to the attention of the authors: where it says “28% had been diagnosed with hypertension”, it had said “28 had been diagnosed with hypertension”. The published article has since been corrected. The authors thank you for reading this erratum and apologize for any inconvenience caused.

